# Dr. Conquest's Outlines of Midwifery

**Published:** 1821-06-01

**Authors:** 


*Outlines of Midwifery, developing its Principles and Practice; with Twelve Lithographic Engravings.* By J. T. Conquest, M. D. F. L. S. Member of the Royal College of Physicians of London; Physician-Accoucheur to the City of London Lying-in-Institution, &c. One vol. small octavo, pp. 193, with twelve engravings, explanations, &c. London, December 1820.

Within these "Outlines," narrow as they are, there is compressed an astonishing mass of elementary and practical matter. Our author tells us, in his preface, that

"The following pages have been written under the abiding and powerful influence of the sagacious reply which was made by a Spartan king, who when asked, 'what is it in which youth ought to be instructed?' promptly answered, 'that which they have most need to practise when men.'" *Preface.*

Our author observes, that an outline of the principles and practice of modern midwifery, in a small compass, and to which reference might be easily made in the study or lying-in-room, has been long wanting; and this has been his inducement to compile the present work.

"The work is avowedly an outline, and not a system; and consequently cannot be intended to supersede more laborious treatises. No lengthened details, contested points, or argumentative disquisitions, are admitted, because it aims at being plain and practical; and from a conviction that a remembrancer is not less useful and necessary than an instructor, it is intended as much for those who require to be reminded as informed. It labours to condense within very narrow limits a large quantity of useful knowledge; and to illustrate the subject of which it treats, rather by a rejection of what is useless, and which ought to be forgotten, than by solicitous inquiries after new materials, that it may be an acceptable compendium for students, or for those young practitioners whose engagements do not permit them to consult more voluminous publications. Still the author has striven not to omit any one really important principle or leading point of practice, which an accoucheur may require for his guidance when at the bed-side of his patient." vi.

The work is strictly obstetric, rejecting all irrelevancies, yet embracing every essential circumstance that can come under the cognizance of a medical man, while conducting a woman through the interesting process of utero-gestation and parturition.

So condensed an elementary and practical work admits of no attempt at analysis; and therefore we can only extract a few passages, and present a few specimens of the matter and manner.

Our first extract shall be from the section on *protracted labours;* and we fear our author, even in these enlightened days, has not much overcharged his sketches.

"Within the whole range of obstetric science, there is nothing which so much distinguishes the judicious practitioner from the man who disgraces medicine, as the management of *protracted labours.* One man, by incessant meddling, produces rigidity of parts, and even inflames the os uteri so that his patient through his folly shall suffer from a most painful and protracted labour.

"Another, officiously interferes with the beautifully simple and admirably adapted process of nature; and presumes that, by rupturing the membranes as soon as he can detect them, or by using his lever on lever principles, by which many women are rendered wretched for life, he shall accelerate parturition.

"A third, urges his patient to be constantly taking stimulants, such as wine and spirits; or to employ voluntary exertion, under the *cant terms* of holding in her breath and forcing down; whilst the os uteri is not dilated half enough to permit the head to be forced through, and the consequence is, that the woman becomes so exhausted by useless exertions, that she at last has not power enough to expel the child, and instruments must be had recourse to.

"Another practitioner allows the head to remain through hours and days of laborious parturition in a position which will never permit it to pass through the pelvis, until the mother is worn out by fruitless efforts; though the malposition might have been rectified at the commencement of labour without difficulty.

"A fifth, is altogether unconcerned about the condition of parts, until the head has been so long, and so firmly wedged in the superior aperture of the pelvis, that mortification follows.

"To complete this mournful series of portraits, every one of which is drawn from a living character, *another*, instead of waiting for uterine action to throw off the placenta, will pull at the funis as at a cart rope, until the uterus is inverted, or formidable hæmorrhage follows; and when, as a consequence of his meddling, the uterus is filled with coagulated blood, and it strives to empty itself by strong contractions, which are called after pains, he will strive to counteract the salutary operation, by exhibiting large doses of opium to quiet these pains, which are intended to repair the mischief he had himself produced. These sketches have not one shade too deep, and they are but a sample of those practical evils, which are of almost every day occurrence." 77.

We observe at page 167, that Dr. Conquest speaks in high terms of the long forceps, as an "invaluable instrument," "but little known and much less estimated, or it would be employed by accoucheurs as a most important substitute for the perforator and crotchet, in many of those cases in which children are now destroyed."

"This instrument is principally applicable,

"*First*, to those cases of deformity at the brim of the pelvis, in which the diminished capacity of the pelvis is from sacrum to pubes, and yet so slight, that a little power beyond what the uterus can employ, would expel living children, which are now almost universally sacrificed at the shrine of prejudice. It is applicable,

"*Secondly*, to those cases of hæmorrhage, convulsions, &c. in which the head of the child is resting on the superior aperture of the pelvis, and in which delivery being essential to the well doing of the mother, is now usually effected by opening the head of the child.

"The *long forceps* in contra-distinction to the *short ones*, are to be applied over the occiput and face of the child, so that the convex edges of the blades may correspond to the concavity of the sacrum.

"When used, the power may be exerted laterally, or from side to side, with moderate traction, remembering that the axis of the brim of the pelvis requires the handles to be kept backwards towards the os coccygis, but as the head descends, its most favourable position in relation to the pelvis must be secured.

"It has been extremely gratifying to *myself and to several highly esteemed friends*, to have been by this means instrumental already in rescuing not a few children whose heads had been condemned to be opened." 108.

The preferable mode of procuring premature labour, where that event is desirable, consists, our author states, in gently carrying the forefinger of the left hand through the os uteri, and then passing it round and round within the os and cervix urteri, so as to detach the *decidua*. By this mode the membranes are left entire, so that the fœtus cannot be destroyed by pressure, (as is the case where the membranes are ruptured) and the mouth of the womb and vagina are gradually dilated by the protrusion of the liquor amnii performing its wedge-like office, as in natural labour. Parturition usually commences in from twenty-four to ninety-six hours, and the management of the case depends on the nature of the presentation.

On the subject of spontaneous evolution of the fœtus, our author thinks that it is rather "a doubling of the fœtus, so that the arm changes its situation but very little, perhaps not at all, whilst the nates are forcibly expelled before the upper extremity." Our readers will see that this corresponds with Dr. Gooch's ideas, in our last number.

The section on uterine hæmorrhage contains a great mass of important, judicious, and valuable matter. We must close this article with the following extract:—

"The exhibition of very large doses of *opium*, to restrain uterine hæmorrhage, has been recommended by several deservedly eminent accoucheurs.

"Both reason and experience appear to concur in condemning this practice; for whilst it is admitted that under some circumstances opium is highly beneficial, its indiscriminate employment is undoubtedly fraught with mischief.

"The result of calm and dispassionate investigation on this subject is, that opium in large doses, in cases of uterine hæmorrhage, generally does harm, by paralising the contractile energies of the uterine and arterial fibres; and that this valuable medicine is useful, and only useful under the existence of some such circumstances as the following:

"It is decidedly beneficial, when hæmorrhage has gone on until the vital powers have become reduced extremely low; and when, with other symptoms of exhaustion, the stomach manifests great irritability.

"It is no less valuable an agent, when hæmorrhage is the consequence of irregular contraction of the uterine fibres, whether of the circular or longitudinal.

"In either of these cases, it is a very efficacious article of the materia medica; but it appears most dangerous to attempt to maintain its utility, or to rely on its efficacy in cases of active and alarming uterine hæmorrhage.

"When exhibited under the before mentioned circumstances, to secure its full effect, it is necessary to give it in doses of four or five grains, repeating it every second or third hour whilst necessary, with a diminution of one grain from each successive dose." 169.

The foregoing extracts do not do justice to the little volume before us; and any attempt at a regular analytical delineation would be doing it a still greater injustice. We remember well the time when, in the early years of our obstetric practice, we should have considered this pocket reference, on puzzling occasions, in the lying-in-room, a most welcome treat. As such we recommend it to the young practitioner; while to the more experienced accoucheur it will prove a useful remembrancer.

